# Uncommon Mechanism of Mangled Lower Limb Trauma: Case Report of Salvaged Leg Post Gear Box Explosion Injury

**DOI:** 10.1002/ccr3.72567

**Published:** 2026-04-27

**Authors:** Khalifa Al Alawi, Iman Zaher, Asim Al Baraznji, Ali Darwiche, Mohammed Muneer

**Affiliations:** ^1^ Department of Plastic & Reconstructive Surgery Hamad Medical Cooperation Doha Qatar; ^2^ Department of Physiotherapy Hamad Medical Cooperation Doha Qatar; ^3^ Department of Orthopaedic Surgery Hamad Medical Cooperation Doha Qatar

**Keywords:** bone transfer, free flap, lower limb trauma, rehabilitation

## Abstract

Mangled limb injuries, typically caused by high‐energy trauma, present significant challenges in surgical decision‐making, particularly when considering limb salvage versus primary amputation. While common mechanisms include vehicular or industrial accidents, unconventional etiologies are rarely documented. We report a unique case of a 43‐year‐old female who sustained a mangled left lower limb injury following a gearbox explosion in a Porsche Cayenne. The injury resulted in a near‐complete amputation at the distal third of the leg, with extensive contamination by oily and metallic debris. Radiologic assessment confirmed complex fractures and vascular injury, with transection of the posterior tibial and peroneal arteries. Despite a high Mangled Extremity Severity Score (MESS) of 7 and initial surgical consensus favoring below‐knee amputation, the patient and her family strongly advocated for limb preservation. The initial management involved damage control surgery with extensive debridement and external fixation. A multidisciplinary team provided staged reconstruction despite significant soft tissue loss and neurological deficits. The patient demonstrated favorable vascular recovery and maintained foot viability, ultimately preserving the limb. This case highlights an unusual mechanism of mangled limb trauma and underscores the critical role of shared decision‐making in trauma care. It reinforces that, in carefully selected patients, limb salvage may be feasible even in scenarios traditionally warranting amputation.

## Key Clinical Message

- This case highlights a rare mangled limb injury from a gearbox explosion. Despite an initial indication for amputation, the patient's preference led to a multidisciplinary limb salvage approach. Collaborative decision‐making and coordinated care enabled functional recovery, emphasizing the impact of patient autonomy and team‐based management in complex trauma cases.

## 1 Introduction

Mangled limb injuries are among the most challenging conditions to manage in trauma care. A mangled limb typically results from high‐energy trauma, with severe disruption of muscle, bone, nerve, and vascular structures [1]. These injuries often stem from motor vehicle accidents, occupational and industrial incidents, and severe falls. Depending on the injury mechanism, patients may present with either an isolated mangled limb or as part of a polytrauma [2, 3].

Initial management should follow Advanced Trauma Life Support (ATLS) principles, prioritizing patient survival over limb preservation. In the acute setting, surgeons are often confronted with the time‐critical decision between limb salvage and primary amputation. This decision is guided by the extent of osseous and soft‐tissue damage, the mechanism of injury, the anticipated functional outcome after salvage, and the patient's overall physiological status. Importantly, decision‐making should be holistic and incorporate patient‐centered factors such as the patient's values and preferences, age, and occupation [3, 4].

We present a case of a mangled lower limb caused by an unusual mechanism of trauma.

## 2 Case Presentation/Examination

A 43‐year‐old Egyptian female with no medical or surgical history presented to Hamad General Hospital with a left lower‐limb injury following a gearbox explosion in her 2008 Porsche Cayenne. On arrival, she was hemodynamically unstable and was initially managed by the trauma team according to ATLS protocol.

**FIGURE 1:**
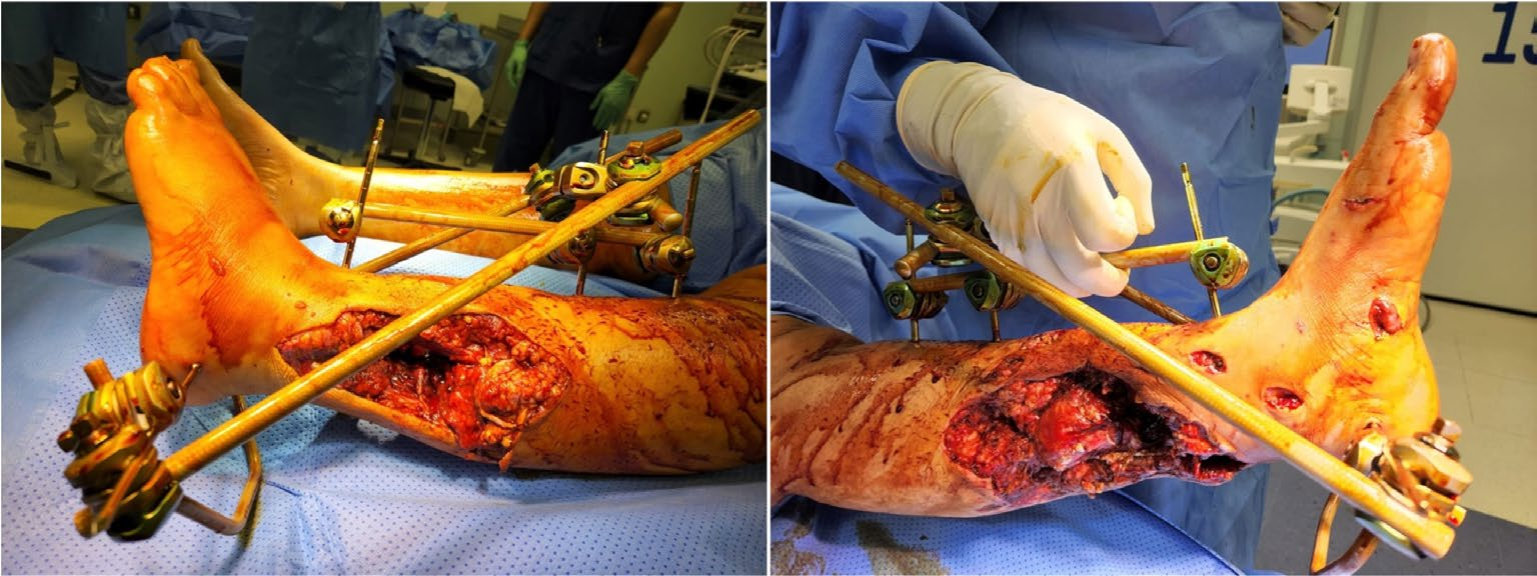
Left leg distal third wound with near amputation with part of anterior and posterior skin intact.

On physical examination, the left leg was nearly amputated at the distal third, with only the anterior skin bridge remaining intact (Figure 1). The wound was contaminated with black oily material and metallic debris. No peripheral pulses were palpable, and capillary refill was markedly sluggish. A neurological assessment was not possible due to intubation. Radiographs showed a comminuted fracture of the distal tibia and fibula, with metal fragments embedded in the soft tissue and foot. CT angiography revealed a patent anterior tibial artery, but transection of the posterior tibial and peroneal arteries. A pan‐CT scan excluded other injuries.

### 2.1 Deferential Diagnosis

Not applicable.

### 2.2 Management

Initially, the patient underwent damage‐control surgery. The wound was debrided, foreign bodies were removed, and a triplane external fixator was applied to stabilize the fracture. Steinman pins were placed proximally and distally, and the foot was protected from pressure ulcers with a U‐shaped rod. The wound was dressed, and the patient was transferred to the trauma ICU, where she required transfusion of 2 units of blood.

The next day, the patient was extubated and reported decreased sensation over the lateral and dorsal aspects of the left foot, with inability to extend the toes. Vascular examination showed normal capillary refill. A multidisciplinary team, including orthopedic and plastic surgeons, evaluated the patient. In light of a high Mangled Extremity Severity Score (MESS) of 7 and severe bone loss and soft‐tissue damage, the team initially recommended below‐knee amputation. However, after shared decision‐making with the patient and her family, she declined amputation and requested limb salvage.

**FIGURE 2:**
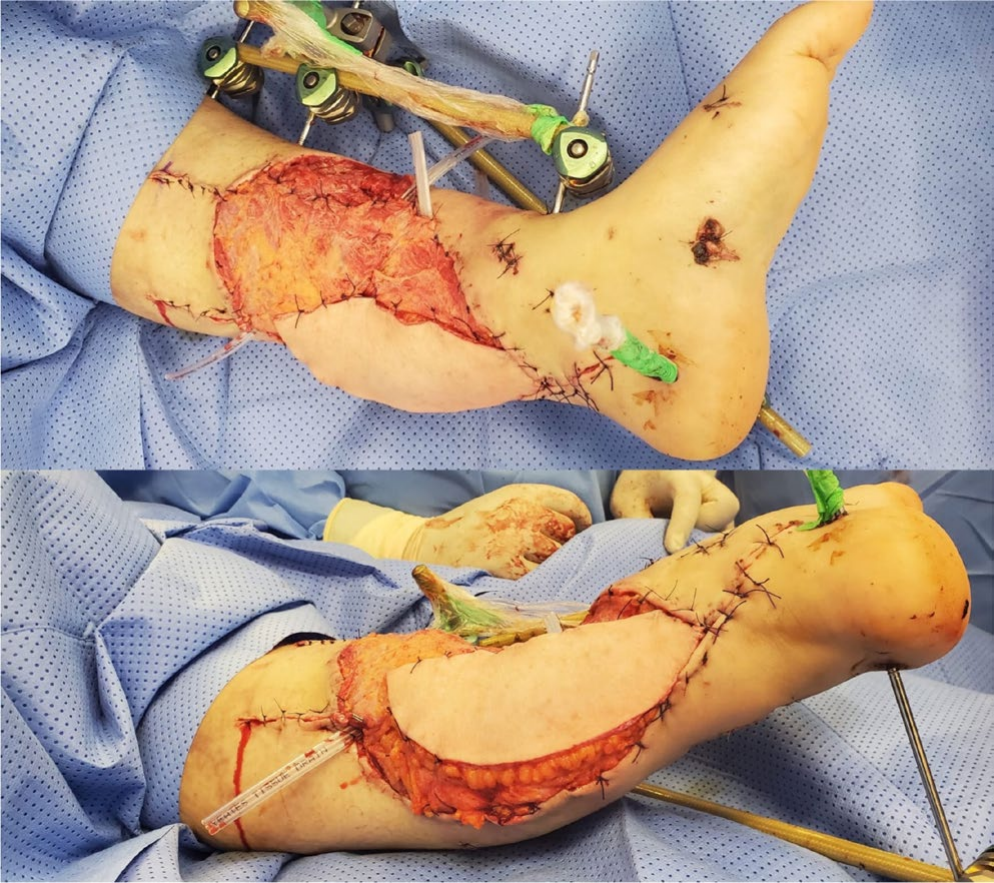
Left lower limb defect covered with LD flap.

Two days later, in line with the patient's preference for salvage, she underwent a second debridement and vacuum‐assisted closure (VAC). A third‐stage debridement was conducted with the plastic surgery team, which revealed transection of the posterior tibial nerve and artery. Two days later, soft‐tissue reconstruction was performed using a latissimus dorsi free flap (Figure 2). During the postoperative period, the flap was complicated by distal necrosis, which was debrided and covered with a split‐thickness skin graft.

**FIGURE 3:**
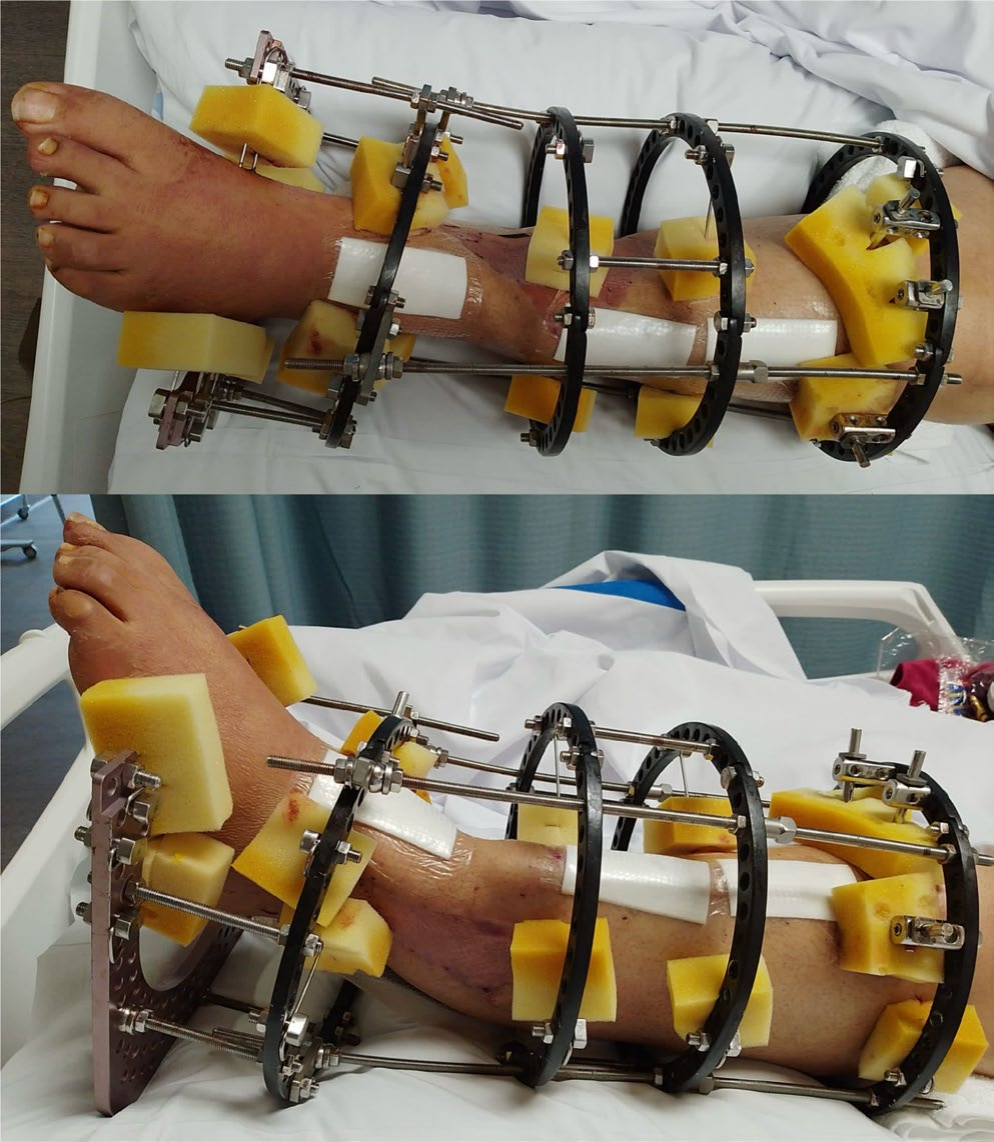
Left lower limb with Ilizarov frame.

After 6 weeks, examination revealed healed skin grafts and a well‐settled flap; however, the patient remained unable to dorsiflex her toes and had altered sensation on the plantar aspect of the foot. At approximately two and a half months, the external fixator was replaced with an Ilizarov frame, and bone transport was performed to address the tibial defect. The procedure involved bone transfer from the proximal tibia to the distal tibial defect, with correction of the foot deformity (Figure 3).

Postoperatively, the patient remained hospitalized for 7 months for wound care and rehabilitation. Twelve weeks after the last surgery, she began weight‐bearing on the Ilizarov frame, with improving sensation and toe movement. The patient was discharged and followed as an outpatient for wound care and physiotherapy. One year later, the Ilizarov frame was removed. The patient started to ambulate with an Aircast boot and subsequently developed bowing at the bone transport site. An intramedullary nail was later inserted to correct the deformity.

### 2.3 Surgical Technique for Ilizarov Bone Transport

It is crucial to perform thorough debridement until only viable soft tissue remains. Prophylactic local antibiotics (e.g., antibiotic beads or powder) may be used; in the presence of infection, they provide high local concentrations while limiting systemic exposure. Muscle flap coverage may be performed before, during, or after bone transport; in our case, it was completed before the transport procedure.

**FIGURE 4:**
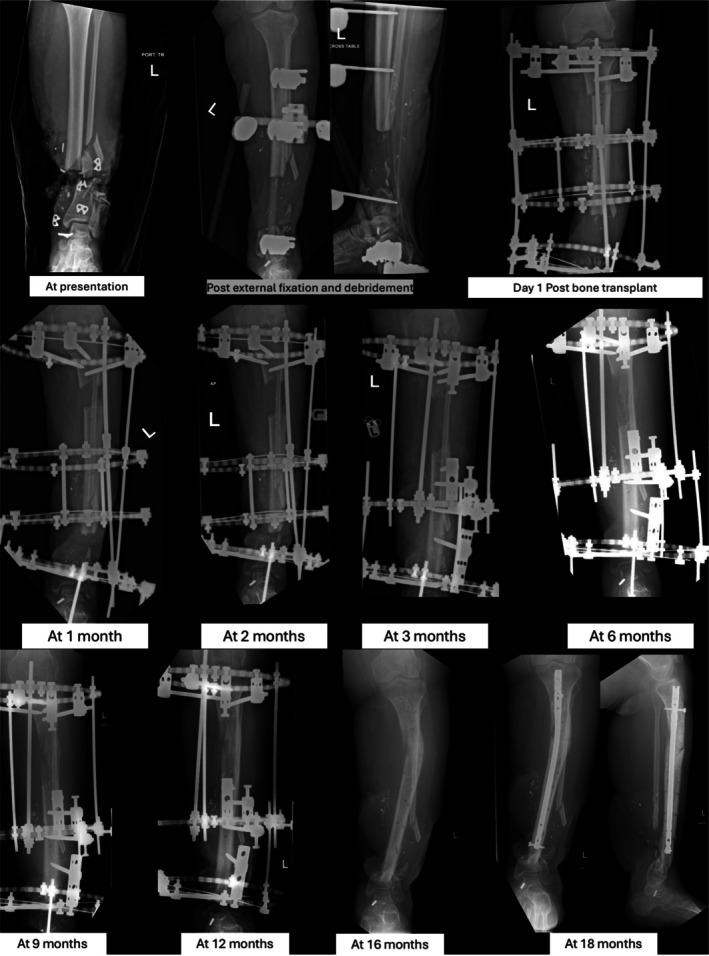
Serial Xray images showing bone transport process with Ilizraov frame.

The Ilizarov frame was applied using four rings: the first at zone 1 (proximal tibia), the second at zone 3 (proximal diaphysis), the third at zone 5 (tibial shaft proximal to the fracture), and the fourth at zone 7 (distal tibia near the ankle, distal to the fracture site). Wires/pins were inserted accordingly, including distal fixation through the calcaneus and metatarsals. After securing the frame, a proximal tibial corticotomy/osteotomy was performed just distal to the tibial tubercle. Once alignment was confirmed, the frame was fully tightened and the construct stabilized (Figure 4).

After a latency period of 10–14 days, bone transport can be initiated in either the inpatient or outpatient setting. The transported segment is advanced toward the defect at a rate of approximately 1 mm/day until docking is achieved. The Ilizarov frame is removed once radiographs confirm consolidation at both the docking site and the regenerate.

### 2.4 From Disability to Ability: A Journey of Hope With Physical Therapy Rehabilitation

The patient was assessed in the Trauma Intensive Care Unit (TICU) following the limb‐salvage operation and received her first physiotherapy (PT) session. She reported severe pain (numerical rating scale [NRS] 8/10). The upper limbs had normal range of motion (ROM), muscle strength, and sensation. In contrast, the lower limbs showed restricted ankle ROM, and muscle strength was graded as 3−/5 at the hips, knees, and great toe extensors; ankle strength could not be assessed. Sensory evaluation, performed by the occupational therapist using a monofilament test, demonstrated reduced sensation (6/10 points using 4.0 and 300 g), with absent sensation in specific areas on the plantar surface of the foot. The patient was unable to sit or stand, and her Functional Ambulation Category (FAC) was 0/5.

Short‐term goals were to maintain and improve muscle strength and joint ROM, promote early mobility, and prevent complications. The long‐term goal was to achieve independent community ambulation (FAC 5/5) within 9 months.

The patient received daily PT sessions that included active ROM exercises for the upper limbs, active‐assisted ROM for the lower limbs, breathing exercises, bed mobility training, sitting/standing practice, and gait training. Despite severe burning pain and numbness, she tolerated the sessions with patient‐controlled analgesia (PCA) boluses as needed. The rehabilitation protocol was adjusted after each procedure; for example, after skin grafting, lower‐limb exercises were withheld for 10 days.

Initially, the patient performed non‐weight‐bearing (NWB) ambulation on the left lower limb with maximal assistance, progressing to minimal assistance. After removal of the external fixator and application of the Ilizarov frame for bone transport, she progressed to full weight‐bearing (FWB) ambulation. During the first week, tolerance was limited and ambulation required maximal assistance with a Canadian frame for support.

One month after Ilizarov frame application, she was able to participate in PT twice daily and attend the gym for stair training, leg press, and balance and gait exercises. Her walking ability improved, progressing from a rollator to elbow crutches.

At discharge, she was medically stable and able to sit, stand, and ambulate independently with elbow crutches (FAC 4/5). The upper limbs and right lower limb demonstrated full strength (5/5), whereas the left lower limb was 3+/5 with restricted ankle and knee ROM. She was advised to continue PT and was provided with a home exercise program, in addition to outpatient PT sessions.

As an outpatient, she attended PT twice weekly until removal of the Ilizarov frame, after which she achieved FWB ambulation using an Aircast boot. She demonstrated improved knee ROM, muscle strength, and balance, progressing to ambulation with a single elbow crutch. However, she developed a complication—bowing at the bone transport site—and subsequently underwent tibial intramedullary nailing (IMN). Following IMN, she resumed full weight‐bearing and continued rehabilitation using the same PT protocol.

## 3 Outcome and Follow Up

**FIGURE 5:**
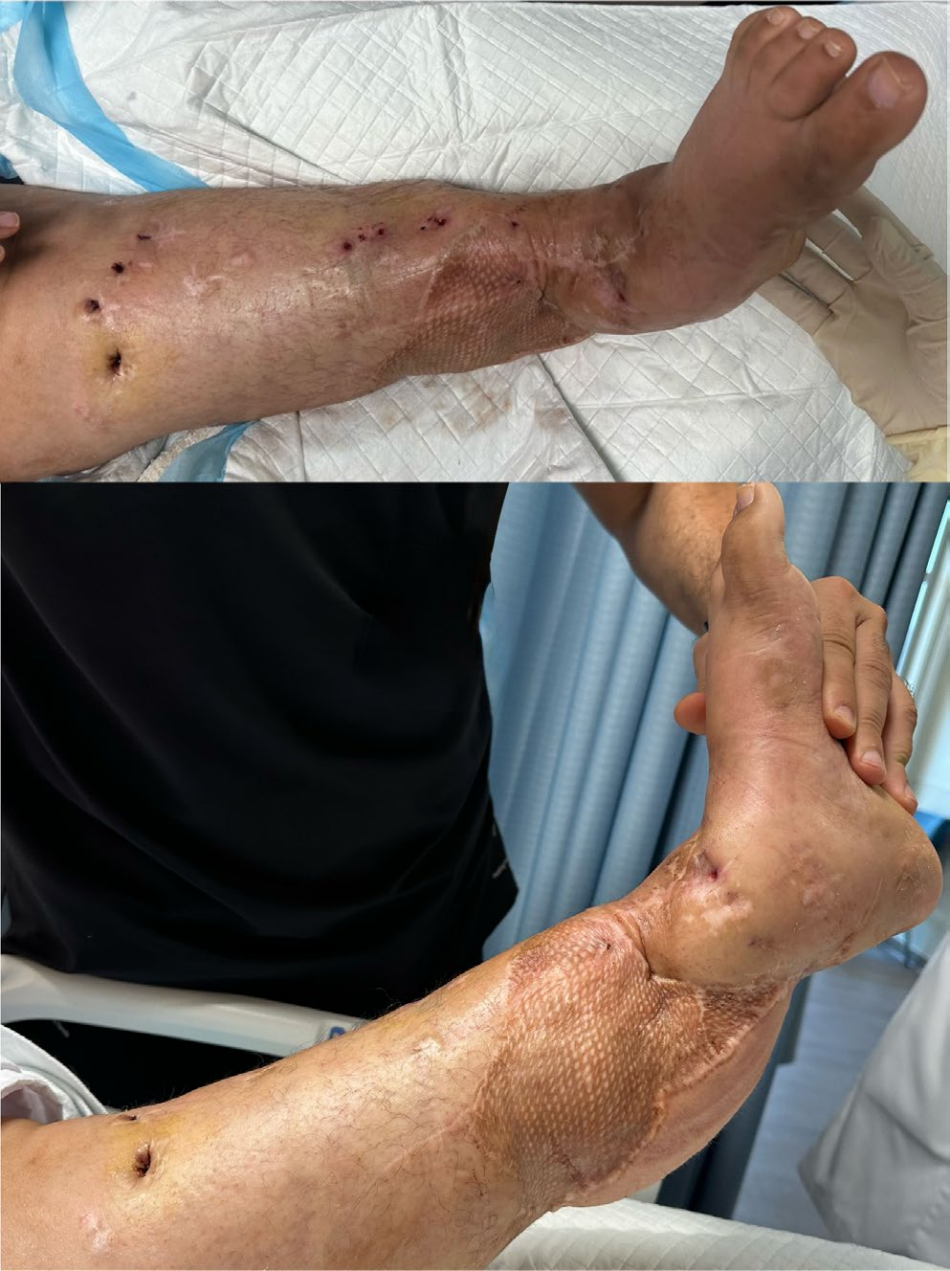
18 months post injury, the images show well settled flap.

At nearly 2 years after the injury, the patient demonstrates significant functional improvement. She is independent in activities of daily living and ambulates without an assistive device; she has also returned to driving. Although she has not yet returned to work or resumed gym‐based exercise, she has expressed strong motivation to pursue these goals. This reflects a marked psychological recovery and a positive outlook, with active plans to take on new tasks and further enhance her independence and quality of life (Figure 5).

## 4 Discussion

Despite the high morbidity associated with motor vehicle collisions, it is exceedingly rare to encounter trauma caused by the explosion of a vehicle component in the absence of an accident. A case has been reported from China, in which a patient sustained bilateral lower limb injuries following the spontaneous explosion of a tire while riding a bus, as she was seated directly above it. She suffered an open right ankle fracture with partial cancellous bone loss, which was managed with debridement, external fixation, and a reverse sural flap [5]. In contrast, the injury in our case was more severe, involving a longer segment of bone loss, vascular disruption, and extensive soft tissue damage, necessitating more complex and prolonged management and rehabilitation.

The decision to pursue limb salvage or proceed with amputation is extremely challenging, particularly in trauma cases. It depends on multiple factors, including the extent of injury, available facilities and surgical expertise, patient preferences, and expected functional outcomes. Therefore, this decision should be guided by a comprehensive assessment that extends beyond intraoperative findings alone [6, 7, 8].

Several scoring systems have been developed to aid in the decision‐making process regarding salvage versus amputation of mangled limbs. Notable examples include the Mangled Extremity Severity Score (MESS) and the Ganga Hospital Open Injury Severity Score (GHOISS) [4, 9]. The MESS was later modified to create the NISSSA score, which incorporates assessments of nerve injury, ischemia, soft tissue injury, skeletal injury, and age. A comparative study evaluating the validity of the NISSSA score against the MESS found that the NISSSA score is both more sensitive (81.8% versus 63.6%) and more specific (92.3% versus 69.2%) [10]. All currently available scoring systems were developed prior to recent advancements in trauma center structures and surgical techniques in orthopedic, plastic, and vascular surgery. For example, the MESS was developed in 1991 based on a retrospective sample of only 25 patients [11]. Similarly, the MESS was modified into the NISSSA score in 1994, which was based on an analysis of 26 patients [10]. Given that these scoring systems are outdated and were based on relatively small sample sizes, the development of newer systems is warranted. In our case, MESS was utilized to make a decision, the patient scored 7, which is an indication for amputation. With this score, both options of whether to amputate or pursue salvage remain valid according to the literature, as the MESS is not predictive for scores of 6–8 [12] Therefore, the team weighed the patient's and family's preferences and decided to attempt to save the limb. In spite of the fact that even if the salvage limb procedure was successful, the functional outcome might not be satisfactory due to nerve injury and chronic infections that require a long duration of medical therapy or repetitive surgeries [13]. In addition, limb amputation is a significant event for the patient, affecting them both physically and psychologically. Therefore, incorporating the patient and/or their family plays a vital role in their management and their acceptance of the outcome.

There are several bone reconstruction techniques, including allograft, vascularized fibular or iliac grafts, hybrid grafts, extracorporeal devitalized autografts, distraction osteogenesis, the induced‐membrane technique, and bone transport. In this case, we utilized a muscle flap and Ilizarov bone transport due to a critical‐sized bone defect. A critical‐sized defect is defined as any defect that will not heal on its own within the patient's lifetime or as one greater than 20 mm or more than 50% of the cortical circumference. Some studies suggest it refers to wedge fragments involving 50%–99% of the cortical circumference without a defined length or more than 50% of the cortical circumference [14, 15]. Our patient had a bone loss of approximately 8.5 cm.

The Ilizarov bone transport technique combines the benefits of external fixation and distraction osteogenesis with healthy soft tissue coverage [16]. It is a useful method for addressing large bone defects and can be applied to both infected and non‐infected tibial bone defects of critical size. Studies have shown that it yields excellent outcomes, with a low rate of poor functional or bone‐related results [17]. One disadvantage of this method is the extended time required for external fixation, and the most common complications include joint stiffness and equinus deformity [18, 19]. Despite these drawbacks, the method has demonstrated a very high union rate, nearly 100%, and a low incidence of reoperation or infection. Additionally, it has shown a high return‐to‐work rate, quicker time to full weight‐bearing, and a reduced need for narcotics [16].

## Author Contributions

**Khalifa Al Alawi:** methodology, supervision, writing – original draft, writing – review and editing. **Iman Zaher:** methodology, writing – original draft, writing – review and editing. **Asim Al Baraznji:** writing – original draft, writing – review and editing. **Ali Darwiche:** project administration, writing – review and editing. **Mohammed Muneer:** project administration, writing – original draft, writing – review and editing.

## Funding

The authors have nothing to report.

## Consent

Written informed consent was obtained from the patient to publish their personal photograph and investigation images in the manuscript.

## Conflicts of Interest

All authors declare no conflicts of interest.

## Data Availability

The data that support the findings of this study are available from the corresponding author upon reasonable request.
